# Supplementary material for: Social Interaction, Noise and Antibiotic-Mediated Switches in the Intestinal Microbiota
Source: PLoS Comput Biol. 2012 Apr 26;8(4):e1002497. doi: 10.1371/journal.pcbi.1002497 (PMC3343147; doi:10.1371/journal.pcbi.1002497)
Supplement: Text S1 — reports additional calculations, figures and details on: 1) model and relative stability analysis, 2) effect of random fluctuations and noise-induced dynamics and 3) Singular Value Decomposition. (PDF) [file pcbi.1002497.s001.pdf]

Social interaction, noise and antibiotic-mediated switches in the intestinal microbiota

Vanni Bucci and Serena Bradde
Program in Computational Biology, Memorial Sloan-Kettering Cancer Center, New York, U.S.A.

Giulio Biroli
Institut Physique Théorique (IPhT) CEA Saclay,
and CNRS URA 2306, 91191 Gif Sur Yvette, France

Joao B. Xavier
Program in Computational Biology, Memorial Sloan-Kettering Cancer Center, New York, U.S.A.

I. MODEL AND STABILITY ANALYSIS

A. Four-dimensional model

We determine the expressions of four biologically meaningful rest points $(\boldsymbol{\rho}_0, \boldsymbol{\rho}_1, \boldsymbol{\rho}_2, \boldsymbol{\rho}_3)$ by setting to zero the right-hand sides of eq. (6) in the main text. The first fixed point $\boldsymbol{\rho}_0 = (1, 0, 0, 1)$ is the one where both bacterial groups are extinct. The second fixed point $\boldsymbol{\rho}_1 = (\mu_s, (1 - \mu_s)/\epsilon, 0, 1)$ represents the sensitive monoculture where $\mu_s = -(a\epsilon)/(\epsilon - m_s)$ and $\mu_t = a/(m_t - 1)$ are the break-even concentrations of ρ_s and ρ_t without antibiotic presence [1]. $\boldsymbol{\rho}_1$ exists if i) $m_s > \epsilon$ and ii) $m_s > \alpha\epsilon$. The third fixed point $\boldsymbol{\rho}_2 = (\mu_t, 0, 1 - \mu_t, 1)$ represents the scenario of tolerant monoculture. This point exists if: i) $m_t > 1$ and ii) $m_t > \alpha$. The last fixed point $\boldsymbol{\rho}_3 = (\mu_s, \rho_{s_3}, \rho_{t_3}, 1)$ corresponds to the coexistence and the relative bacterial density are:

$$
\begin{aligned}
\rho_{s_3} &= \frac{1}{\psi}\left(\frac{m_t}{m_s}\,\epsilon - 1\right) \\
\rho_{t_3} &= \frac{m_s}{m_t}\left(\frac{a}{\epsilon - m_s} + \frac{1}{\psi} + \frac{1}{\epsilon}\right) - \frac{\epsilon}{\psi}\,.
\end{aligned} \tag{1}
$$

Three conditions are necessary for the positivity of $\boldsymbol{\rho}_3$: i) $m_s > \epsilon$, ii) $\epsilon\, m_t > m_s$ and iii) $\psi < \frac{\epsilon(\epsilon - m_s)(\epsilon m_t - m_s)}{m_s(\epsilon\alpha - m_s)}$. Since the parameters are positive, condition iii) gives the additional constrain of $m_s > \epsilon\alpha$.

The stability of the system is studied by linearising eq. (6) of the main text around each of the four fixed points and studying the sign of the eigenvalues of the relative Jacobian matrix, which is defined by:

$$
\mathbf{J} = \begin{bmatrix}
-\frac{a(m_s\rho_s + m_t\rho_t)}{(a+S)^2} - 1 & -\frac{m_s}{a+S}S & -\frac{m_t}{a+S}S & 0 \\
\frac{a m_s}{(a+S)^2}\rho_s & \frac{m_s}{a+S}S - \epsilon & 0 & 0 \\
\frac{a m_t}{(a+S)^2}\rho_s & -\psi\rho_t & \frac{m_t}{(a+S)}S - \psi\rho_s - 1 & 0 \\
0 & 0 & 0 & -1
\end{bmatrix}. \tag{2}
$$

The eigenvalues relative to $\mathbf{J}_{\boldsymbol{\rho}_0}$ are $\lambda^0_1 = -1, \lambda^0_2 = -1, \lambda^0_3 = -\frac{m_t}{a+1} - 1, \lambda^0_4 = -\frac{m_s}{a+1} - \epsilon$. $\boldsymbol{\rho}_0$ is stable if all eigenvalues $\boldsymbol{\lambda}^0$ are negative, which determines the following inequalities: i) $m_t < \alpha$ and ii) $m_s < \alpha\epsilon$. It is worth noticing that the conditions ensuring the stability of $\boldsymbol{\rho}_0$ are the opposite of those for the existence of $\boldsymbol{\rho}_1$ and $\boldsymbol{\rho}_2$.

The stability of $\boldsymbol{\rho}_1$ is determined by studying the sign of: $\lambda^1_1 = -1, \lambda^1_2 = \frac{\psi(\mu_s - 1)}{\epsilon} + \frac{\mu_s m_t}{a+\mu_s} - 1, \lambda^1_{3,4} = -\sigma \pm \sqrt{\sigma^2 - \frac{4\epsilon a^3 m_s^3(\epsilon(a+1) - m_s)}{(\epsilon - m_s)^3}}$, where $\sigma = \frac{a m_s[a m_s + (\epsilon(a+1) - m_s)(\epsilon - m_s)]}{2(a+\mu_s)^2(\epsilon - m_s)^2}$. The imposition of $\lambda^1_2 < 0$ gives the following inequalities: i) $m_s > \epsilon$ and ii) $m_s/m_t > \frac{\epsilon^2}{\psi(1-\mu_s)+\epsilon}$. The conditions for $\lambda^1_{3,4} < 0$ are equivalent to those for $\boldsymbol{\rho}_1$ existence. In summary, if $\boldsymbol{\rho}_1$ is well-defined, it is stable given the condition ii).

The eigenvalues associated to $\mathbf{J}_{\boldsymbol{\rho}_2}$ read: $\lambda^2_1 = -1, \lambda^2_2 = -1, \lambda^2_3 = m_s/m_t - \epsilon, \lambda^2_4 = -\frac{(m_t-1)^2 - a(m_t-1)}{a m_t}$. The conditions for the stability of $\boldsymbol{\rho}_2$ are: i) $m_t > \alpha$ and ii) $m_s/m_t < \epsilon$.

To study the stability of $\boldsymbol{\rho}_3$, we use the Routh-Hurwitz criteria [4]. Let $p = r^4 + c_1 r^3 + c_2 r^2 + c_3 r + c_4$ being the fourth-order characteristic polynomial for $\mathbf{J}_{\boldsymbol{\rho}_3}$, then the rest point $\boldsymbol{\rho}_3$ is stable given the necessary and sufficient conditions: i) $c_1 > 0$, ii) $c_3 > 0$, iii) $c_4 > 0$ and iv) $c_1c_2c_3 > c_3^2 + c_1^2 c_4$. It is easy to verify that conditions i) and ii) are always satisfied:

$$
c_1 = \frac{(\epsilon + m_s)}{m_s} + \frac{(\epsilon - m_s)^2}{m_s} > 0
$$

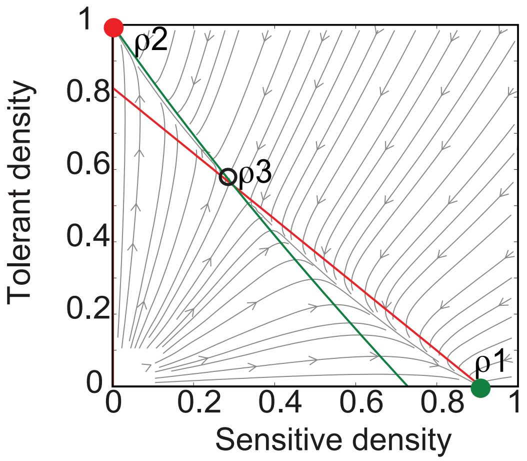

FIG. S1: Vectorial field of forces and the phase-plane analysis for bistable conditions, for the following parameter values: ratio between tolerant and sensitive maximum growth rate $f = 1.1$, antibiotic killing rate $\epsilon = 1.1$ and social interaction rate $\psi = 0.7$. We draw the three rest points $\boldsymbol{\rho}_1$ (blue circle), $\boldsymbol{\rho}_2$ (red circle) and $\boldsymbol{\rho}_3$ (empty circle), where $\boldsymbol{\rho} = (\rho_s, \rho_t)$ is the vector having for components the sensitive s and tolerant t densities, and the system nullclines defined by $d\rho_s/dt = 0$ (red line), $d\rho_t/dt = 0$ (blue line) whose intersection individuate the saddle unstable rest point $\boldsymbol{\rho}_3$.

$$c_3 = \frac{a}{(a+\mu_s)^3}[m_s\rho_{c_3} + m_t\rho_{p_3} + m_s m_t \psi \rho_{c_3}\rho_{p_3}] > 0$$

However, it is also fairly easy to see that condition iii) does not hold. Given the expression for c_4

$$c_4 = \frac{\epsilon\rho_{c_3}(\epsilon - m_s)^2[m_s(1+\psi\rho_{c_3}) - m_t(\epsilon + \psi\rho_{p_3})]}{a m_s^2},$$

condition iii) requires that $m_s(1+\psi\rho_{c_3}) > m_t(\epsilon + \psi\rho_{p_3})$. After some algebra we can see that this condition is false when the rest point is well-defined.

It is now possible to determine the criteria describing the system mono- or bistability in function of the model parameters. By assuming the existence of both fixed points and by comparing the conditions of stability obtained from the linearisation analysis we derive the following relationships:

- Monostability with only sensitives $\frac{m_t}{m_s}\epsilon < 1$,
- Monostability with only tolerants $\frac{m_t}{m_s}\epsilon > 1 + \frac{\psi}{\epsilon}(1-\mu_s)$,
- Bistability with both mutually exclusive sensitives and tolerants monocultures $1 < \frac{m_t\epsilon}{m_s} < 1 + \frac{\psi}{\epsilon}(1-\mu_s)$.

These criteria highlight two major concepts. First, it is necessary to have a negative feedback (i.e. $\psi > 0$) from sensitives to tolerants for bistability to arise. If no negative feedback is present the system can set only in one of the two mono-stable states. Second, the modulation effect of the antibiotic ϵ. It is clear that an increase in antibiotic-killing needs to be counteracted by an increase in selective pressure in order to maintain sensitives stability.

B. Two-dimensional model

The two dimensional model of eqs. (1) and (2) in the main text is obtained by: 1) substituting eq. (7) of the main text into eq. (6), 2) simplifying the saturation terms by dividing numerator and denominator by m_s and 3) introducing $f = m_t/m_s$.

We repeat the linear stability analysis and we determine three equivalent fixed points (Fig. S1): $\boldsymbol{\rho}_1 = (1/\epsilon, 0)$, which represents the sensitive monoculture, $\boldsymbol{\rho}_2 = (0, 1)$, which represents the tolerants monoculture and $\boldsymbol{\rho}_3 = (\frac{\epsilon f - 1}{\psi}, \frac{\psi + \epsilon(1-\epsilon f)}{\psi\epsilon f})$ which represents a state where both groups coexist. $\boldsymbol{\rho}_1$ and $\boldsymbol{\rho}_2$ are always exist while state $\boldsymbol{\rho}_3$

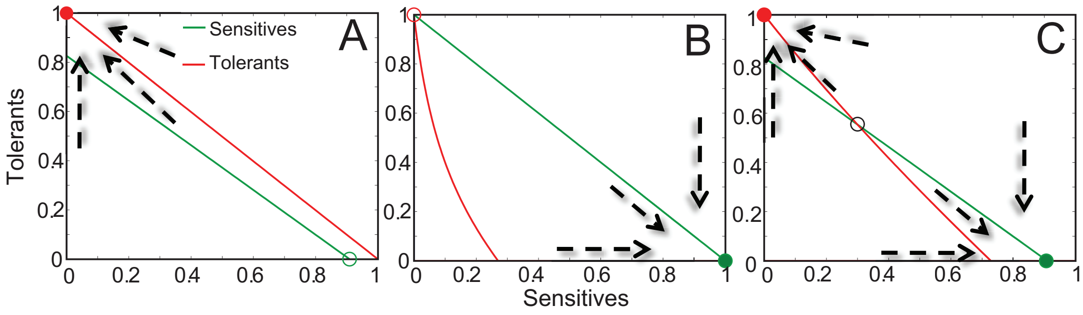

FIG. S2: Model nullclines analysis in the absence of noise. A: the tolerants nullcline lies above the sensitives nullcline leading to tolerants dominance and sensitive extinction. The corresponding parameter set is $f = 1.1, \epsilon = 1.1, \psi = 0.1$. B: the sensitives nullcline lies above the tolerants nullcline leading to sensitives dominance and tolerants extinction. The corresponding parameter set is $f = 1.0, \epsilon = 1.0, \psi = 5$. C: the tolerants nullcline is steeper than the sensitives nullcline and their intersection is a saddle and unstable point. The stable manifold of the saddle divides the interior of the quadrant into the sets of initial conditions leading to competitive dominance by one type of microbe and competitive exclusion of the other. The corresponding parameter set is $f = 1.1, \epsilon = 1.0, \psi = 0.7$.

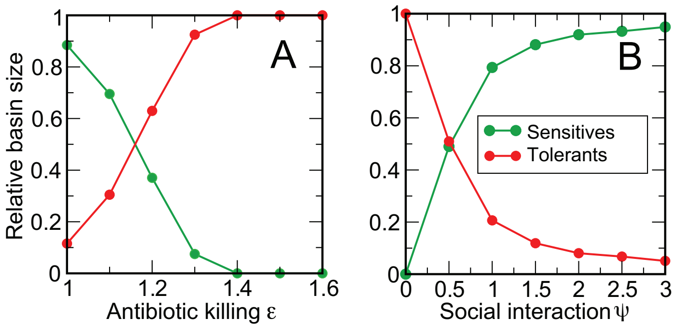

FIG. S3: Normalized-to-one areas of the basins of attraction, corresponding to sensitives (green curve) and tolerants (red curve), versus the antibiotic-killing ϵ (A) or the social interaction ψ (B).

exists if and only if $1 < \epsilon f < 1 + \psi/\epsilon$. The Jacobian matrix $\mathbf{J}(\boldsymbol{\rho})$ now reads:

$$\mathbf{J}(\boldsymbol{\rho}) = \begin{bmatrix} \frac{f\rho_t}{(\rho_s+f\rho_t)^2} - \epsilon & -\frac{f\rho_s}{(\rho_s+f\rho_t)^2} \\ -\psi\rho_t - \frac{f\rho_t}{(\rho_s+f\rho_t)^2} & \frac{f\rho_s}{(\rho_s+f\rho_t)^2} - \psi\rho_s - 1 \end{bmatrix}. \quad (3)$$

$\boldsymbol{\rho}_1$ has eigenvalues $\lambda_1 = -\epsilon$ and $\lambda_2 = \epsilon f - \psi/\epsilon - 1$. Thus, since ϵ is positive-defined, $\boldsymbol{\rho}_1$ is stable if and only if $\epsilon f < 1 + \psi/\epsilon$. Equivalently, eigenvalues in $\boldsymbol{\rho}_2$ are -1 and $1/f - \epsilon$. $\boldsymbol{\rho}_2$ is stable if and only if $\epsilon f > 1$. Since the characteristic polynomial of $\mathbf{J}$ is $p = r^2 + c_1 r + c_2$ the conditions for $\boldsymbol{\rho}_3$ stability are $c_1 = -\lambda_1 - \lambda_2 > 0$ and $c_2 = \lambda_1\lambda_2 > 0$. These conditions are equivalent to verifying that the real parts of λ_1 and λ_2 are strictly negative. The expression for c_1 and c_2 are the following:

$$c_1 = \epsilon f - \frac{\epsilon^2(1-f)(1-\epsilon f)}{\psi}$$

$$c_2 = \epsilon\frac{(1-\epsilon f)}{\psi}[\epsilon(1-\epsilon f) + \psi].$$

The first condition implies that $\psi/\epsilon > (1-\epsilon f)(1-f)/f$. The condition is true only in the particular case when $f > 1$, which by itself does not prove the instability of $\boldsymbol{\rho}_3$. However, in order to have $c_2 > 0$, the argument inside the square bracket has to be negative (i.e. $\psi/\epsilon < \epsilon f - 1$), which is the opposite of the one ensuring $\boldsymbol{\rho}_3$ existence. As a consequence, if $\boldsymbol{\rho}_3$ exists, it will be unstable analogously to the four-dimensional model of the previous section.

The system stability features can be visualized by drawing the system nullclines (i.e. the curves represented by $\frac{d\rho_s}{dt} = 0$ and $\frac{d\rho_t}{dt} = 0$) in the phase-plane defined by tolerant ρ_t vs. sensitive ρ_s densities (Fig. S2). Tolerants domination $\boldsymbol{\rho}_2$ is always obtained for parameter sets resulting in the tolerants nullcline laying above the sensitives one (Fig. S2A) and the reverse is true for sensitives dominance $\boldsymbol{\rho}_1$ (Fig. S2B). Bistability is obtained when the nullclines intersect in the saddle unstable coexistence point $\boldsymbol{\rho}_3$ such that the stable manifold of the saddle divides the interior of the quadrant into the sets of initial conditions leading to competitive dominance by one type of microbes and competitive exclusion of the other. In absence of fluctuations, depending on the system initial conditions, a time-trajectory will be attracted in one of the two mutually exclusive stable states $\boldsymbol{\rho}_1$ or $\boldsymbol{\rho}_2$ where it will persist indefinitely (Fig. S2C). The phase-plane is divided into two attracting basins, one around the tolerant mono-culture and the other around the sensitive mono-culture. Their size can be determined with a Monte Carlo search in the phase space (Fig. S3).

II. NOISE-INDUCED DYNAMICS

The integration of the Langevin dynamics in presence of bistability shows that the system time evolution in the presence of noise is non-trivial. The microbiota switches over-time between the antibiotic-tolerant and the antibiotic-sensitive dominations in a non-deterministic fashion that varies for different realizations of the noise. Additionally, in agreement with experimental observations on the level of isolation of individuals [6, 7], it appears that the time of recovery to sensitive-domination depends on the magnitude of the noise variance (Fig. S4B-D).

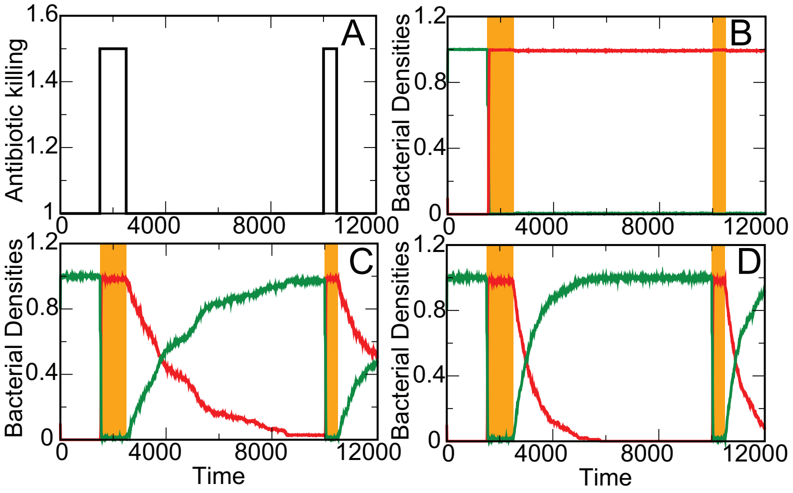

FIG. S4: Time evolution of the sensitive (green) and tolerant (red) densities obtained by solving the Langevin equations for $f = 1.1$, $\psi = 0.7$, ϵ variable with time (see Panel A) and three different noise regimes. A: antibiotic treatment, B: $D \to 0$, C: $D = 0.00033$ and D: $D = 0.001$. The densities are obtained averaging over 100 noise realizations and show the strong dependence of the return to sensitive domination after treatment on the noise level. Orange shaded region represents treatment conditions. The dynamics here shown qualitatively reproduces the behaviour observed in longitudinal microbiome data (see Fig. 5).

We can think that the introduction of the noise leads to a diffusion process within the space of possible microbiota compositions such that the time of escape from each stable or meta-stable state becomes strictly finite. The strength of the diffusive motion is given by the size of the noise variance, D. Increasing D, the system spends a shorter time to wander far from the initial configuration which coincides with the increase of the probability of crossing the attracting basins separatrix in shorter time. Previous studies have characterized the mean residence time in each domination by computing the escape rate between the two stable states, in the limit of small D, in terms of stationary probability distribution [9, 10]. However, in our case this function is not known a priori since the system is non-conservative. Even though alternative numerical solutions (e.g. explicit integration of the Langevin equations or of the Fokker-Planck Equation) can be used to do so, these methods can be numerically very intensive and become prohibitive when the number of states increases (i.e. solving a partial differential equation in $d \gg 3$ dimensions). As a consequence, in the main text we follow a new alternative theoretical framework based on transition state theory.

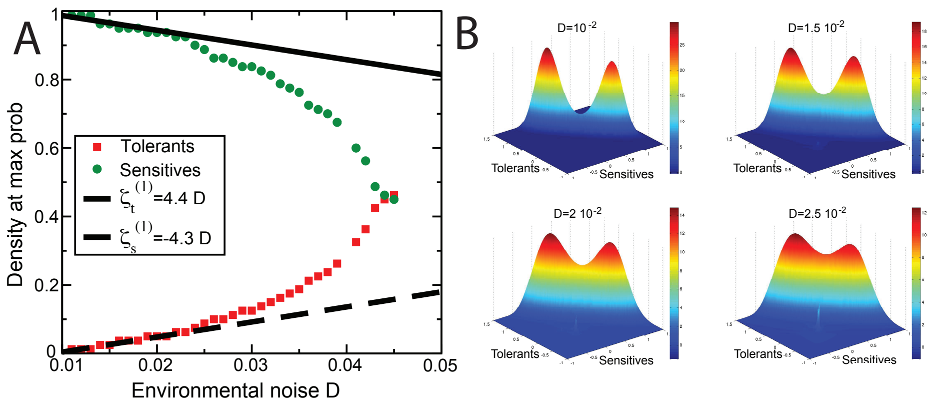

FIG. S5: A: most probable bacterial density $\boldsymbol{\rho}$ change with respect the noise parameter D when the boundary condition are fixed at negative values far enough from the location of the stable states. The set of parameters used is $f = 1.1, \epsilon = 1.1$ and $\psi = 0.4$. This configuration is not physical since we allow negative values of the densities. However, we show that the theoretical prediction of the linear coefficients reported in the main text (see Results section) coincides with the numerical simulation here reported. B: plot of four different stationary distributions for $f = 1.1, \epsilon = 1.1, \psi = 0.4$ and $D = 0.01, 0.015, 0.02, 0.025$ obtained solving numerically the FPE with the following boundary conditions: $P^s(-1, \rho_t) = 0$ and $P^s(\rho_s, -1) = 0$.

Numerical estimates of the mean residence time

In order to characterize the stochastic dynamical behaviour of the bacterial concentrations we can numerically estimate the moments of their joint probability distribution ($P(\boldsymbol{\rho})$) by sampling different possible trajectories connecting the two stable states multiple times. Each time-trajectory is obtained by solving the Langevin equations with different realizations of the noise ($\boldsymbol{\xi}$) using a Milstein integration scheme [8]. In the main text, we compare the estimate of the residence time in each domination state (t_i with $i = 1, 2$) obtained with this sampling technique with that determined using the novel theoretical framework.

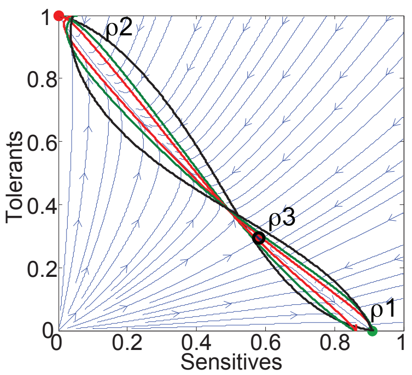

FIG. S6: Stationary path connecting the stable points 1 and 2. The red, green and black solid curves are the trajectories associated with different values of the initial velocity, 0.032 0.072 and 0.172 respectively showing that the most probable path for small noise is concentrated along the unstable manifold as obtained for different sampled trajectories (data not shown). It is worth emphasizing that for conservative fields of forces, meaning $\mathbf{F} = -\boldsymbol{\nabla} U$, it easy to verify that $\dot{\boldsymbol{\rho}} = \pm\mathbf{F}$ are both the uphill and downhill optimal path. The solution with the plus sign has null action $\mathcal{S} = 0$ meaning that its probability is equal to unity for every value of the noise D. This means that the path is always deterministic: it describes a simple gradient descent that takes place even in absence of noise. On the contrary the $\dot{\boldsymbol{\rho}} = -\mathbf{F}$ is associated to the reverse path and has a finite action $\mathcal{S} > 0$ meaning it is activated only in presence of noise since its probability is suppressed and has strictly null value when $D = 0$. The optimal path connecting two stable states is formed by an ascending trajectory toward the unstable point, given by $\dot{\boldsymbol{\rho}} = -\mathbf{F}$, followed by a descending trajectory given by $\dot{\boldsymbol{\rho}} = \mathbf{F}$. In presence of a non-conservative force, the scenario changes completely and the uphill and downhill trajectory are different since $\dot{\boldsymbol{\rho}} = -\mathbf{F}$ is no longer a solution of the optimal path equation any longer.

III. SUPPORTING FIGURES FOR SVD

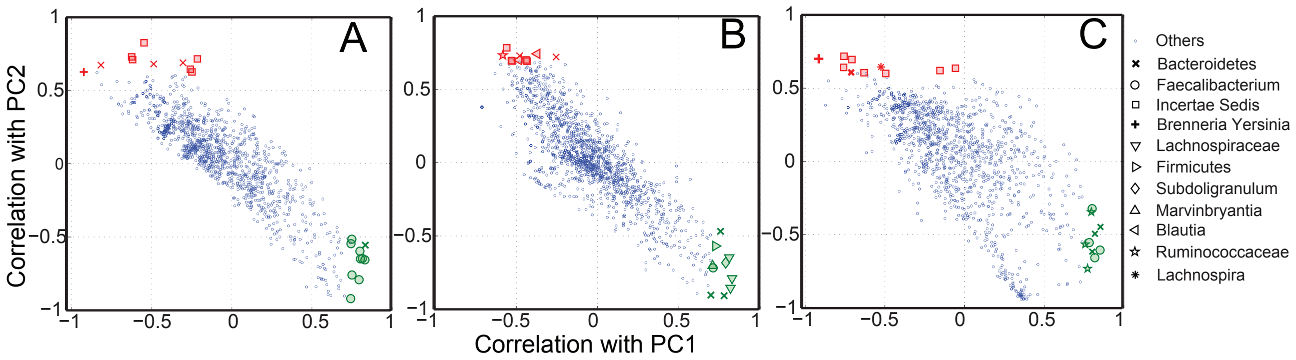

FIG. S7: Plot of the correlation with principal component 2 (PC2) versus correlation with principal component 1 (PC1) for all the phylotypes detected in each subjects (A-C) from [2]. Green (red) are the top 10 most correlated phylotypes with PC1 (PC2) which significantly decrease (increase) in response to antibiotic treatment.

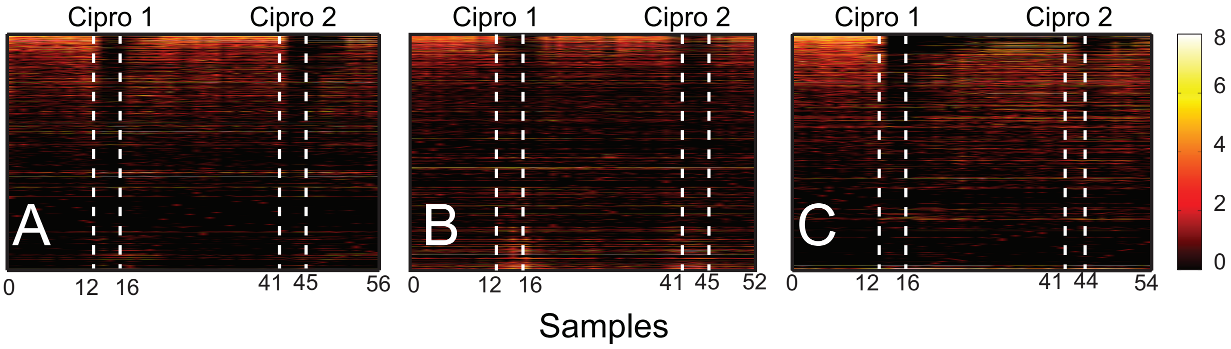

FIG. S8: Log_2 abundance versus samples for all the phylotypes detected in each subject (A-C) from [2] sorted from the most to least correlated with PC1. At the top we individuate the most sensitive phylotypes to antibiotic (mostly decreasing in density) while at the bottom the most tolerant ones (mostly increasing in density). Differently from Fig. 5 in the main text, where only the top 20 sensitives and tolerants are shown, here we display all the detected phylotypes.

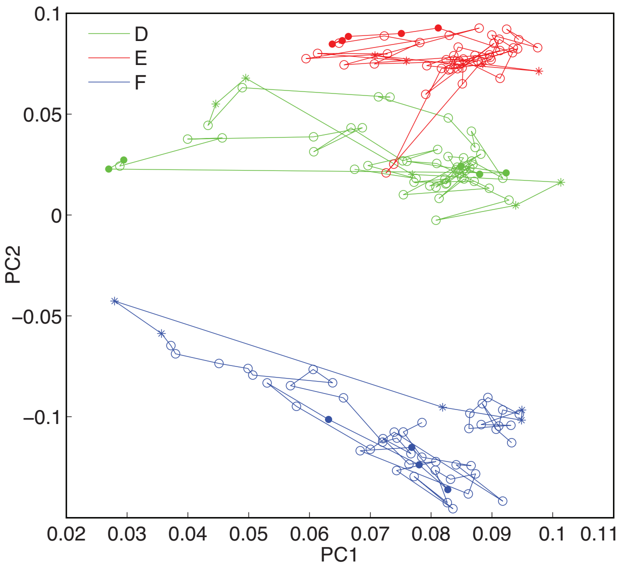

FIG. S9: Ordination plot of the time samples based on their first two principal components. We can easily recognize the time points belonging to the three individuals (inter-individual variability) and their evolution in response to treatment. Empty circles represent untreated samples, asterisks represent samples during treatment 1 and filled circles represent represent samples during treatment 2.

[1] Hsu SB, Li YS, Waltam P (2000) Competition in the presence of a lethal external inhibitor. *Math Biosc*, 167:177-199.
[2] Dethlefsen L, Relman DA (2011) Incomplete recovery and individualized responses of the human distal gut microbiota to repeated antibiotic perturbation. *Proc Natl Acad Sci U S A*, 108 Suppl 1:4554-4561.
[3] Alter O, Brown PO, Botstein D (2000) Singular value decomposition for genome-wide expression data processing and modeling. *Proc Natl Acad Sci*, 97(18):10101-10106.
[4] Otto S, Day T (2007) *A Biologist's guide to mathematical modeling* Princeton, NJ: Princeton University Press.
[5] Fay TH, Joubert SV (2010) Separatrices, *Inter. Journ. Math. Educ. Scie. Tech.* 41(3):412-419.
[6] Ubeda C et al (2010) Vancomycin-resistant enterococcus domination of intestinal microbiota is enabled by antibiotic treatment in mice and precedes bloodstream invasion in humans. *J Clin Invest*, 120(12):4332-4341.
[7] Littman DR, Pamer EG (2011) Role of the commensal microbiota in normal and pathogenic host immune responses. *Cell Host & Microbe*, 10(4):311-323.
[8] Higham DJ (2001) An algorithmic introduction to numerical simulation of stochastic differential equations. *SIAM Rev.*, 43:525-546.
[9] Gardiner CW (1983) The escape time in nonpotential systems. *J. Stat. Phys.*, 30(1):157-177.
[10] Borgis D, Moreau M (1990) On the escape rate from a metastable state in a non-potential system. *Physica A* 163:877-894.
[11] Gardiner CW (1997) Handbook of stochastic methods, Third Edition, eds Springer series in Synergetics (Spinger Hedeilberg).
[12] Mao-Jones J, Ritchie KB, Jones LE, Ellner SP (2009) How microbial community development composition regulates coral disease development. *Plos Bio.*,8(3):e1000345. doi:10.1371/journal.pbio.1000345
[13] Seifert U (2008) Stochastic thermodynamics: principles and perspectives.*EPJ B*, 64(3-4):423-431.
[14] Langer JS (1967) Theory of condensation point. *Ann Phys* 41:108-157
[15] Langer JS (1968) Theory of nucleation rates. *Phys Rev Lett*, 21(14):973-976.
[16] Sidney Coleman (1988) Aspects of symmetry, eds Cambridge University Press.
